# STAKEHOLDERS’ PERSPECTIVES ON AN AREA AGENCIES ON AGING SERVING URBAN OLDER ADULTS

**DOI:** 10.1093/geroni/igad104.0010

**Published:** 2023-12-21

**Authors:** Faith Hopp, Fay Keys

**Affiliations:** Wayne State University, Detroit, Michigan, United States; Wayne State University, Detroit, Michigan, United States

## Abstract

Area Agencies on Aging (AAAs) in urban areas play a critical role in helping older adults maintain and enhance their quality of life. The Detroit Area Agency on Aging (DAAA), recognizing the importance of involving Detroit area seniors and community stakeholders in long-term strategic initiatives, funded a comprehensive needs assessment of the region’s seniors. This study reports findings from an online survey in the winter of 2020, (n=94) aimed at identifying stakeholder views of DAAA programs and services. We used descriptive statistical analyses for closed-ended items and content analysis to identify themes from open-ended questions. Most respondents were either direct service purchase vendors (34.0%) or contractors (22.3%). The highest ranked perceived agency role was information and assistance (mean=2.41; SD=1.02), followed by direct service provision (mean=2.45; SD=1.52), coordination of aging services (mean=2.63; SD=1.54), education (mean=3.40; SD=1.11), and training (mean=4.10; SD=0.99). The most commonly mentioned areas needed for the region to become age-friendly were supportive community services (66.3%), public transportation (44.9%), safety and security (41.6%), available and affordable housing (40.4%), access to healthcare (38.2%), and housing maintenance/modification (24.7%). The most common themes from open-ended responses included outreach, collaboration, and promoting aging in place. These findings suggest the imperative of providing accessible, high-quality services that promote aging in place in urban areas through community outreach and collaboration activities. Detroit area seniors are a vital community resource, and ongoing stakeholder input is needed as part of collaborative efforts to meet their complex emerging health and social needs.

